# Fault detection in switching process of a substation using the SARIMA–SPC model

**DOI:** 10.1038/s41598-020-67925-3

**Published:** 2020-07-10

**Authors:** Guo-Feng Fan, Xiao Wei, Ya-Ting Li, Wei-Chiang Hong

**Affiliations:** 1 School of Mathematics & Statistics, Pingdingshan University, Pingdingshan, 467000 China; 2 Department of Information Management, Oriental Institute of Technology, Panchiao, New Taipei, Taiwan

**Keywords:** Energy science and technology, Engineering, Mathematics and computing

## Abstract

To detect substation faults for timely repair, this paper proposes a fault detection method that is based on the time series model and the statistical process control method to analyze the regulation and characteristics of the behavior in the switching process. As the first time, this paper proposes a fault detection model using SARIMA, statistical process control (SPC) methods, and 3σ criterion to analyze the characteristics in substation’s switching process. The employed approaches are both very common tools in the statistics field, however, via effectively combining them with industrial process fault diagnosis, these common statistical tolls play excellent role to achieve rich technical contributions. Finally, for different fault samples, the proposed method improves the rate of detection by at least 9% (and up to 15%) than other methods.

## Introduction

The substation equipment is the hard-hub of a power system, and ensuring its safe and reliable operation is very important^1^. Voltage transformers’ faults, capacitors’ faults, and bus-bars’ faults can cause long-term failure of a substation. These causes of substation failures are characterized by diversity and randomness, which make detecting substation equipment faults very difficult^2^. One effective way to detect substation equipment faults is to analyze the regulation of behavior in the switching process of a substation. Therefore, switching process behaviors are deserved to be investigated.

Methods for detecting substation equipment faults currently fall into two categories—static detection and dynamic detection. Methods in the first category detect power failure that is caused by a faultless trip of a substation switch. Such methods only solve the problem of large-scale power failure of a substation because the operations of the system in a substation are inactive, protection device does not provide any action signals, and monitoring device is not assigned a scheduled trip. These methods cannot detect small-area power failures in a timely manner^3–5^. Dynamic detection is a method of continuous detection using assistant facilities, such as the substation direct current (DC) power supply online loop alarm system, which can identify faults with power. Dynamic detection is guaranteed by establishing multiple transmitting substations, which wirelessly connect to nearby faulty probes and then transmit a fault signal to the global system for mobile (GSM)-receiving website over a GSM network. However, the GSM system is limited by its capacity, poor switching function, and terminal-accessing rate^6^. To improve the accuracy and the stability of the switched positive linear systems, Yin et al.^7^ presented three advanced algorithms that impose some requirements on the switching signal to obtain a tighter bound on the average dwell time (ADT). Liu et al.^8–10^ and Chang et al.^11^ proposed a series of innovative quantization algorithms for hybrid control to increase the stability and detection accuracy of Takagi–Sugeno nonlinear systems. Since a substation is a classical digital system, other factors, such as quantization of the transmission signal, may also influence system performance and the accuracy of fault detection. Xiong et al.^12^ proposed a fault detection filter for uncertain dynamic linear systems with quantization to guarantee asymptotic stability and fault detection accuracy. Vazquez et al.^30^ presented an expert system for diagnosis of power system fault allocation in real time (SIDUF-TR). The system used information on the tripped relays and circuit breakers to identify the most probable faulted element of the power system, serving as a decision-making support for energy control center dispatchers. Huang et al.^31^ proposed a new method based on improved empirical mode decomposition (EMD) energy entropy and multi-class support vector machine (MSVM) to diagnose fault for high voltage CB.

In recent years, the statistical process control (SPC) method for monitoring production and operation processes has been extensively investigated with the goal of improving product quality^12–14^. The SPC method is based on a hybridization of statistics and engineering, comprehensively considering the processes of change in state space and time dimensions. It not only overcomes the inherent shortcoming of the static detection method, which is its lack of timely detection, but also facilitates the extraction of the characteristics of dynamic changing fault signals, ultimately improving the effect of fault detection. The basic theoretical premise of the regular control chart is that observed sample data are mutually independent and subject to fit the same distribution^15^. However, most controlled processes in production do not meet these conditions. For example, in the metallurgical, chemical, and electronic industries, all production processes are continuous, and data are automatically collected, so the sampling interval is relatively short, and observations (sample data) are likely to suffer from auto-correlation^16–18^. A regular control chart cannot properly perform the functions in production processing controls with auto-correlated sample data^19–21^, and extensive evidence has demonstrated that a residual control chart is more effective with auto-correlation^22–27^.

Given the above, this paper proposes a method for detecting substation faults using the SPC model and the time series model. First, the time series model is used to analyze the characteristics of the switching behaviors of a substation, and thus to construct a suitable seasonal time series model, $SARIMA_{{\left( {p,d,q} \right) \times \left( {P,D,Q} \right)}}$, to obtain the independent residual sequences that follow the same distribution. Second, the determined independent residual sequences are used to demonstrate a one-way control chart and a difference analysis is performed, ultimately discriminating the state of the substation^28,29^. This proposed method overcomes the challenges associated with the above conditions and the deficiencies of other methods. It is a simple, reliable, and high-value approach for detecting substation faults.

The motivations for this paper are as follows. First, substation equipment is crucial to any power system, which it protects. Accordingly, the operating status of substation equipment (such as high voltage circuit breakers) should be monitored to detect, diagnose, and troubleshoot various faults in time to minimize losses.

In particular, in the opening and closing operation of high voltage circuit breakers, the current waveform of a DC electromagnetic coil contains a wealth of information, and fault detection by a high-voltage circuit breaker exploits the characteristics of the current signal. According to the current waveform and some characteristics of the current signal, the operators can judge whether the core motion has the phenomenon of sticking, tripping, dividing and closing. In the process of automatic monitoring, fault diagnosis and fault classification of high voltage circuit breakers, the computer is required to automatically obtain the characteristics of the current signal. After extracting the features, the current data is modelled by mathematical statistical method, and a method for detecting substation faults is obtained.

Second, other methods, such as the expert system method^30^, chiefly rely on empirical data to make judgments and if change occurs that it beyond the experience of the experts, their judgment will probably be invalid.

Finally, the SPC method uses statistical tools to identify clues before the fault occurs, and to extract the associate features accurately, increasing detection accuracy. Moreover, based on the waveform of the coil current and some characteristics of the current signal, some intelligent algorithms (such as the multi-class support vector machine (MSVM)^31^) can determine whether the iron core is stuck, has tripped, has been rejected, for example, to identify and diagnose faults. However, owing to the complexity of the combined information and the different scales of information in some characteristics of the sub-health status, the accuracy of detection is not high. In this investigation, three levels of information (which contain different contents) are used to extract separately the features, effectively improving the accuracy of detection, especially when the system is in the sub-health state.

## Results

### Fault detection preparation and implementation

The testing data for the fault detection model are current data obtained during substation switching between 1 and 31st July, 2017. Owing to the implementation of two switching processes (open and closed), four operational statuses and their associated data are collected. These collected data are further separated into four types of sample—normal samples, minor fault samples, moderate fault samples, and serious fault samples. The characteristics during the switching process are extracted from collected historical data of the substation. The sampling points are selected with a certain sampling interval. To eliminate random effects, ten experiments are carried out for each operational status. Data that were collected data from the four operational statuses are used to generate the time series diagram. These data are processed to construct the $ARIMA_{{\left( {0,1,1} \right)}}$ model, and the extracted current data are used to determine the pre-fitting performance. As mentioned above, if the pre-fitting performance is not satisfactory, then the substation is obviously under the failure status, and the system is suffering from serious faults, and is likely to be difficult to recover.

The residual error from data with a satisfactory pre-fitting performance are further processed using the $SARIMA_{{\left( {0,1,1} \right) \times \left( {0,1,1} \right)}}$ model. The results demonstrate that the substation is associated with poor fitting performance and the system has a moderate fault status and can be recovered. This result is consistent with the actual status.

**Figure 1:**
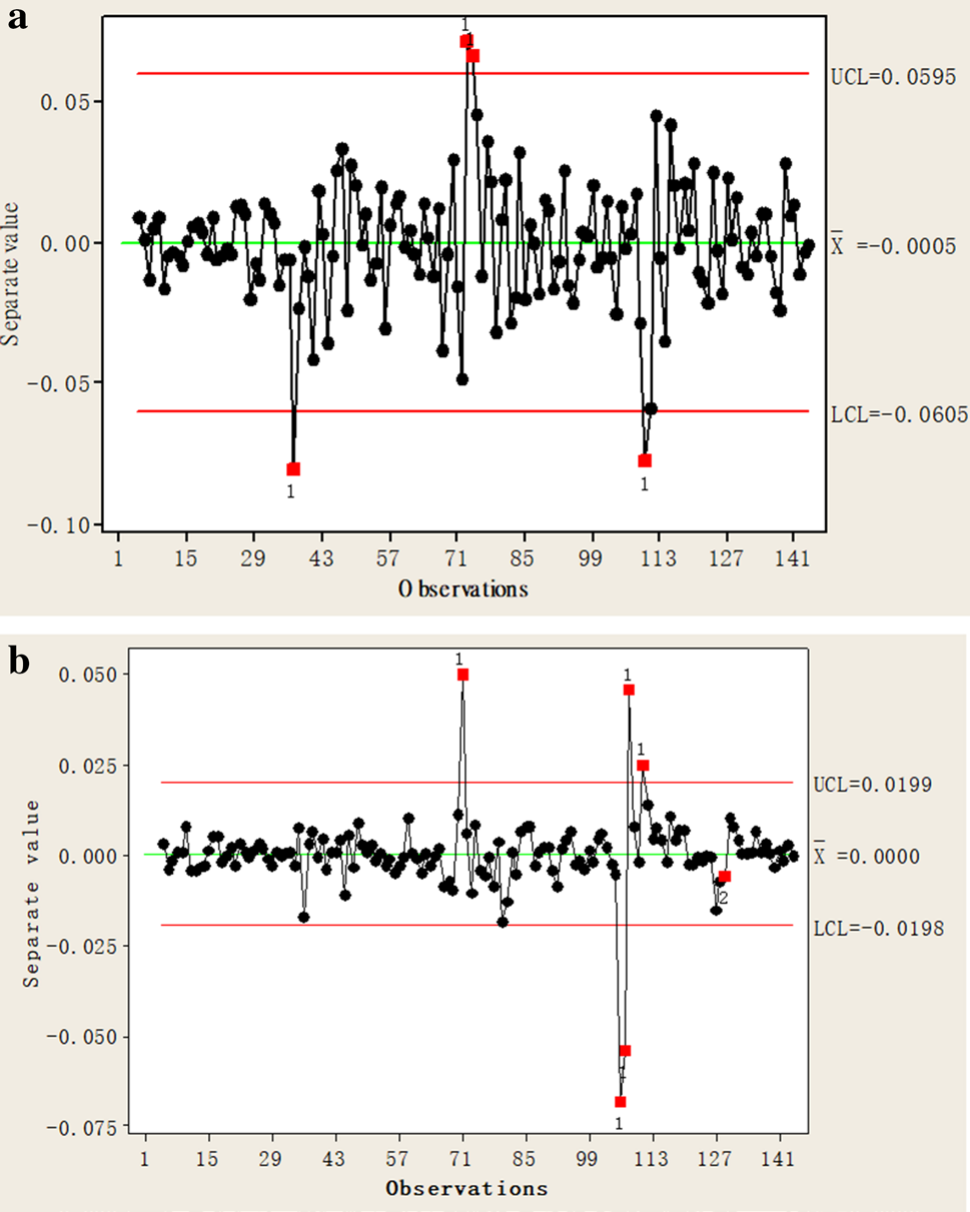
(**a**) One-way control chart of the open process for minor fault samples. (Calculated by Minitab 16.0 version (Minitab Inc.)). (**b**) One-way control chart of the closed process for minor fault samples. (Calculated by Minitab 16.0 version (Minitab Inc.)).

The correlated residual error, $MS_{2}$, from the $SARIMA_{{\left( {0,1,1} \right) \times \left( {0,1,1} \right)}}$ model can be used to generate a one-way control chart of the stationary autocorrelation process, as presented in Fig. 1a,b for the two switching processes, respectively. The results (Figs. 1a,b) of this process are calculated using the statistical software Minitab version 16.0 by Minitab Inc.

Singularity discriminant detection based on the 3σ criterion is conducted. The results demonstrate that the detection point on the control map is not between the UCL and the LCL, so, it is the outlier point, indicating that the substation is under a slight fault status. This detection result is consistent with the actual status, so timely maintenance and repair of the system are required.

Briefly, when the data of the four running states are processed, if the $ARIMA_{{\left( {0,1,1} \right)}}$ model is used to pre-process the extracted current data and get a poor processing effect, the substation is in a serious fault state. Then, the data with good pre-processing effect are further processed with the model after adding seasonal factors. The substation with poor pre-processing effect is in obvious fault state. The sequence of correlation residuals obtained from the established $SARIMA_{{\left( {0,1,1} \right) \times \left( {0,1,1} \right)}}$ model, and the one-way control diagram of the stationary autocorrelation process will be drawn with the residuals. According to the 3$\sigma$ criterion, when the point on the control chart is not between the upper control limit UCL and the lower control limit LCL, it is the unqualified point (the outlier), and the substation is in a sub-health state. Otherwise, the substation is in a healthy state. Suppose the production process is in a normal state, the mean of the population is $\overline{x}$, the standard deviation is $\sigma$, then the positions of the control limit are, $CL = \overline{x}$, $UCL = \overline{x} + 3\sigma$, and $LCL = \overline{x} - 3\sigma$.

Tables 1 and 2 present the statistical results of the $SARIMA_{{\left( {0,1,1} \right) \times \left( {0,1,1} \right)}}$ model for these two processes (open and closed) in different substations under different statuses.

**Table 1:** The analysis of $SARIMA_{{\left( {0,1,1} \right) \times \left( {0,1,1} \right)}}$ model for the open process in different substations.

The substations	Models	Probabilities (*P*_1_ for MA; *P*_2_ for SMA)	Residual error (MS_2_)
A	SMA*	0.007	0.0000349
	MA**	0.006	
B	SMA*	0.000	0.000227
	MA**	0.033	
C	SMA*	0.013	0.0000988
	MA**	0.000	
D	SMA*	0.970	0.0000629
	MA**	0.000	
E	SMA*	0.000	0.000396
	MA**	0.000	
F	SMA*	0.000	0.000448
	MA**	0.000	
G	SMA*	0.000	0.000499
	MA**	0.000	
H	SMA*	0.000	0.0000591
	MA**	0.000	
I	SMA*	0.426	0.000490
	MA**	0.000	
J	SMA*	0.000	0.000461
	MA**	0.000	

*SMA model implies the $SARIMA_{{\left( {0,1,1} \right) \times \left( {0,1,1} \right)}}$ model.

**MA model implies the $ARIMA_{{\left( {0,1,1} \right)}}$ model.

**Table 2:** The analysis of $SARIMA_{{\left( {0,1,1} \right) \times \left( {0,1,1} \right)}}$ model for the closed process in different substations.

The substations	Models	Probabilities (*P*_1_ for MA; *P*_2_ for SMA)	Residual error (MS_2_)
A	SMA*	0.021	0.0000365
	MA**	0.042	
B	SMA*	0.000	0.0000556
	MA**	0.000	
C	SMA*	0.005	0.0000268
	MA**	0.004	
D	SMA*	0.000	0.0000119
	MA**	0.000	
D'	SMA*	0.601	0.0000333
	MA**	0.000	
E	SMA*	0.000	0.0000338
	MA**	0.013	
F	SMA*	0.000	0.0000731
	MA**	0.001	
G	SMA*	0.001	0.000122
	MA**	0.000	
H	SMA*	0.000	0.0000324
	MA**	0.000	
I	SMA*	0.335	0.0000487
	MA**	0.000	
J	SMA*	0.000	0.0000911
	MA**	0.000	

*SMA model implies the $SARIMA_{{\left( {0,1,1} \right) \times \left( {0,1,1} \right)}}$ model.

**MA model implies the $ARIMA_{{\left( {0,1,1} \right)}}$ model.

### Results analysis

Table 1 indicates that in the open process, the test probability, $P_{2}$, exceeds 0.05 in substations ***D*** and ***I***, implying that the $SARIMA_{{\left( {0,1,1} \right) \times \left( {0,1,1} \right)}}$ model does not well fit in the open process for either the ***D*** or the ***I*** substation. This result has two reasons: the first is that this substation switching process is under the failure status in the early stage or under the wear and tear failure status, such as due to a blown fuse, short circuit to ground, line breaks, or an unqualified contact. The second is that the current fluctuations in the open process are differ significantly from others.

Table 2 demonstrates that in the closed process, the test probability, $P_{2}$, exceeds 0.05 in the ***I*** substation. It also implies that the $SARIMA_{{\left( {0,1,1} \right) \times \left( {0,1,1} \right)}}$ model does not accurately fit in the closed process in the ***I*** substation. Two reasons explain this result: the first is that this substation switching process also under the failure status in the early stage or under the wear and tear failure status, such as lead damage conduction resistance. The second is that the current fluctuations in the closed process differ significantly from others.

Comparing the statistical results for the open and closed processes in Tables 1 and 2 demonstrates that the test results from substation ***D*** are inconsistent, but that the relevant data are correct. Therefore, all data obtained during the closed process are sorted in time order and new results are further calculated using the proposed method, the received result is denoted as ***D***'. The new result, ***D***', in the closed process also indicates that the substation is under the failure status, consistent its status in the open process. Thereby, the new result, ***D***', reflects the adaptability of the proposed method.

## Discussions

The proposed method is based on the residual model that is constructed to fit the system closely. The residual errors are further analyzed using the discriminating criterion to identify the faults of a substation. The traditional residual control model may generate a certain error because most diagnostic targets belong to a large power system, and it is not suitable for application to a nonlinear system. However, the method accounts for the fact that the current waveform contains considerable information due to the special structure of the open-closed switching circuit. The status of a switching circuit can be determined by monitoring and analyzing the current waveform; the performance of the entire high voltage circuit breaker can therefore also be predicted. The three models (ARIMA, SARIMA, and SPC models) used herein and the three current regulations are orthogonally decomposed. They are all independent of each other after decomposition, and the information obtained from three current waveforms and the fault statuses are highly accurate.

The accuracy of recognition is compared with those of two other models, the expert system^30^ and the multi-class support vector machine (MSVM)^31^. Table 3 presents some of the detection results.

**Table 3:** The detection results of the $SARIMA_{{\left( {0,1,1} \right) \times \left( {0,1,1} \right)}}$-SPC model and other potential models in different substations.

Sub-stations	The discrimination vector (*P*_1_, *P*_2_, *N*)	Expert system method^30^	MSVM model^31^	$SARIMA_{{\left( {0,1,1} \right) \times \left( {0,1,1} \right)}}$-SPC model	Detection results (1 month later)
A	(0.007, 0.006, 1)	Fault-free	Fault-free	Fault-free	Fault-free
B	(0.000, 0.033, 0)	Fault-free	Fault-free	Fault-free	Fault-free
C	(0.013, 0.000, 3)	Fault	Fault	Sub-health	Operating mechanism stuck
D	(0.601, 0.000, 1)	Obvious fault	Fault	Serious fault	Capacitor fault
E	(0.000, 0.000, 1)	Fault-free	Fault-free	Fault-free	Fault-free
F	(0.000, 0.000, 0)	Fault-free	Fault-free	Fault-free	Fault-free
G	(0.000, 0.000, 5)	Fault-free	Fault-free	Sub-health	Poor contact at the line head
H	(0.000, 0.000, 1)	Fault-free	Fault-free	Fault-free	Fault-free
I	(0.426, 0.000, 2)	Obvious fault	Fault	Serious fault	Circuit breaker refuses to close
J	(0.000, 0.000, 1)	Fault-free	Fault-free	Fault-free	Fault-free

*N* is indicated as the number of 25 consecutive outlier points.

In Table 3, when *N* is greater than or equal to 3, the substation is in sub-health state, particularly, when *N* is equal to 3, there are the following situations: (i) three discrete outliers; (ii) two consecutive outliers; and (iii) three consecutive outliers. Additionally, in Table 3, the differences for different kinds of detection results, such as sub-health, obvious fault, and serious fault are further defined in Table 4.

**Table 4:** The definitions of the detection results.

Detection results	Discrimination criteria	Common faults	Recovery possibility
Serious fault	$P_{1} \notin \left( {0.00,0.05} \right)$	Voltage transformer fault, a current transformer fault, a capacitor fault, the refusal of a circuit breaker to close, bus failure, and others	Recovery is difficult
Obvious fault	$P_{2} \notin \left( {0.00,0.05} \right)$	The faults suffered by the system, including resonance, the system mixed line, the porcelain bottle flicker, and others	Can be restored
Sub-health	UCL-LCL beyond	Stuck mechanism or poor contact, and others	Need timely maintenance
Fault-free	UCL-LCL inside	Normal	Normal

The expert system^30^ is a knowledge-based method for determining the fault status of a substation by using information that is obtained by modeling or signal processing. The method is typically based on the differences among the modeling details, or the presented symptoms, or the qualitative models to conduct the diagnosis works. It overcomes the shortcomings of traditional methods of diagnosing faults in large-scale power systems. However, it has its own weaknesses; for example, it requires a large amount of data to generate a knowledge base of accumulated experience. When the available information is limited such as in substations ***C***, ***D***, and J, its diagnostic performance is not good.

The MSVM model is based on signal processing. It requires that numerical calculations are carried out to process collected data, and it identifies faults from the results. However, since it has extensive hardware requirements, a high accuracy of classification of faults is not guaranteed, as evident herein in substations ***C*** and ***D***.

The proposed model diagnoses the sub-health status of each substation with high accuracy. For example, the other two methods only diagnosed substation ***C*** as faulty, without specifying the level of fault, and the diagnosed result was inconsistent with its true status; they misdiagnosed substation ***G*** as fault-free even though it had a sub-health status and suffered from service failures, such as component fatigue, and had to be repaired. In the short term, this substation ***G*** will suffer from a stuck operating mechanism or poor contact at the line head, and in the long term, an unknown but serious fault will occur.

Table 5 presents the improvement in the efficiency of a well-known electric power company in Henan, China. To clearly demonstrate the detection accuracy calculation, Type I error ($\alpha$) and Type II error ($\beta$) are also considered, i.e., the detection accuracy in Table 5 is the ratio of the correctly estimated samples to the number of samples. It is obtained after the Type I error and Type II error are excluded (as shown in Eq. (1)).

1 $$ Detection \,accuracy = \frac{Total \;samples - \alpha - \beta }{{Total \;samples}} \times 100\% $$

**Table 5:** The 100 detection results of the $SARIMA_{{\left( {0,1,1} \right) \times \left( {0,1,1} \right)}}$-SPC model and other potential models in different substations.

Sub-stations	Expert system model^30^	MSVM model^31^	$SARIMA_{{\left( {0,1,1} \right) \times \left( {0,1,1} \right)}}$-SPC model	The maximum improvements than other models
A	85 (Type I = 11, Type II = 4)	88 (Type I = 8, Type II = 4)	95 (Type I = 3, Type II = 2)	10% (= (95 − 85)/100 × 100%)
B	87 (Type I = 9, Type II = 4)	91 (Type I = 7, Type II = 2)	96 (Type I = 2, Type II = 2)	9%
C	79 (Type I = 15, Type II = 6)	85 (Type I = 8, Type II = 7)	94 (Type I = 4, Type II = 2)	15%
D	83 (Type I = 9, Type II = 8)	82 (Type I = 12, Type II = 6)	97 (Type I = 1, Type II = 2)	15%
E	90 (Type I = 5, Type II = 5)	87 (Type I = 7, Type II = 6)	96 (Type I = 3, Type II = 1)	9%
F	82 (Type I = 11, Type II = 7)	85 (Type I = 9, Type II = 6)	93 (Type I = 4, Type II = 3)	11%
G	85 (Type I = 10, Type II = 5)	88 (Type I = 9, Type II = 3)	96 (Type I = 1, Type II = 3)	11%
H	84 (Type I = 7, Type II = 7)	90 (Type I = 6, Type II = 4)	93 (Type I = 5, Type II = 2)	9%
I	85 (Type I = 7, Type II = 8)	80 (Type I = 12, Type II = 8)	94 (Type I = 3, Type II = 3)	14%
J	80 (Type I = 9, Type II = 11)	92 (Type I = 6, Type II = 2)	95 (Type I = 4, Type II = 1)	15%

Type I error is the sample size or percentage of normal status that are detected as failure status, i.e., the normal processes are misdiagnosed as the abnormal ones.

Type II error is the sample size or percentage of failure status that are detected as normal status, i.e., the abnormal processes are misdiagnosed as the normal ones.

It used the proposed method to increase the accuracy of their circuit breaker failure detection by 9% to 15% in different areas of the country: in the central areas, the increase exceeded 9%; in eastern areas, it was approached 15%. These results demonstrate that the proposed method exhibits good adaptability and effectively identifies faults in substations. The experimental results were obtained using a HZ-101A high-voltage switch characteristic tester.

Finally, to confirm the reliability of the proposed model, *Re*, a reliability calculation method in the quality control field, is used herein. The circuit is a series connection system, i.e., the fault level diagnosis of the proposed model is also a series mode, so the reliability, *Re*, can be calculated using Eq. (2),

2 $$ \begin{array}{*{20}c} {Re = Re_{1} *Re_{2} *Re_{3} } \\ {Re_{1} = 1 - P_{1} } \\ {Re_{2} = 1 - P_{2} } \\ {Re_{3} = \left\{ {\begin{array}{*{20}l} {0.9346} \hfill & {if N = 0} \hfill \\ {1 - {\text{C}}_{25}^{N} \left( {0.9973} \right)^{25 - N} \left( {0.0027} \right)^{N} } \hfill & {other wise} \hfill \\ \end{array} } \right.} \\ \end{array} $$

where $Re_{1}$ denotes the reliability of the $SARIMA_{{\left( {0,1,1} \right) \times \left( {0,1,1} \right)}}$ model without seasonal effects; $Re_{2}$ represents the reliability of the $SARIMA_{{\left( {0,1,1} \right) \times \left( {0,1,1} \right)}}$ model with seasonal effects; $Re_{3}$ represents the reliability of the SPC model; and *N* is the number, 25, of consecutive outlier points, given in Table 3.

**Figure 2:**
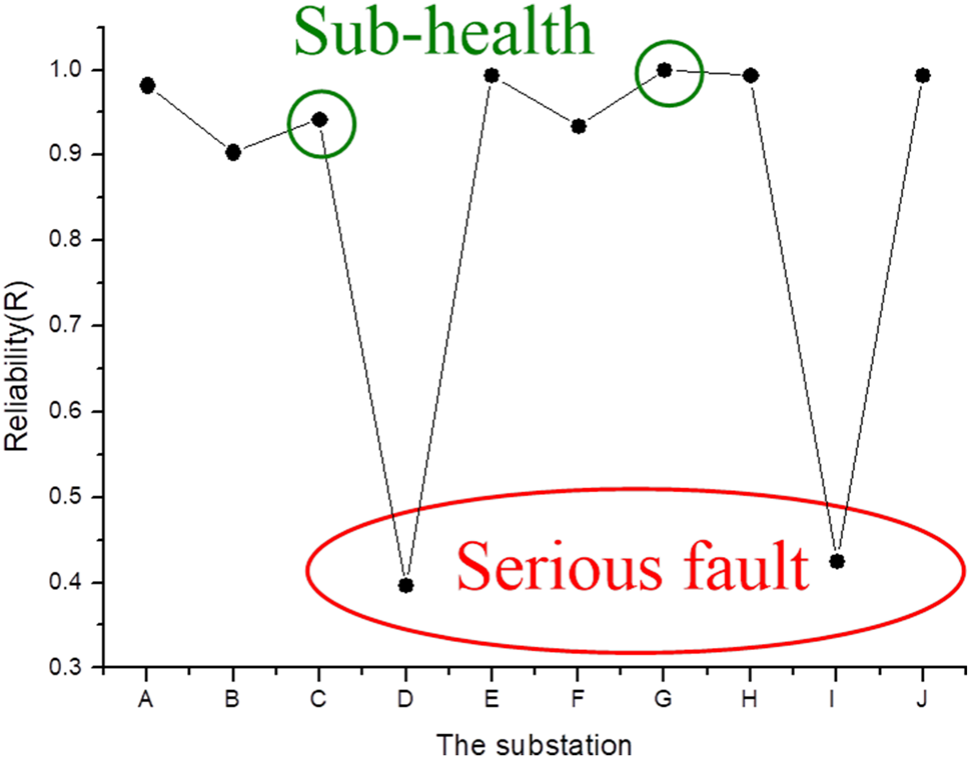
The calculated reliabilities of each substation.

Figure 2 displays the calculated reliabilities of each substation. A red circle implies that the substation is under a serious fault status, such as substations D and I. A green circle indicates that the substation has high reliability, as have substations C and G, but the proposed $SARIMA_{{\left( {0,1,1} \right) \times \left( {0,1,1} \right)}}$-SPC model accurately detects them as having in a sub-healthy status.

This work presents a fault detection method that is based on a time series model and SPC model to detect failure in the two switching processes of a substation. The following conclusions are supported.

1. The proposed method firstly uses the $SARIMA_{{\left( {0,1,1} \right) \times \left( {0,1,1} \right)}}$ model, based on the hardware’s data collection, to detect a fault of a substation. Then, the residual errors of the $SARIMA_{{\left( {0,1,1} \right) \times \left( {0,1,1} \right)}}$ model are used to generate a one-way control chart in the stationary autocorrelation process. Finally, the discriminant detection is applied, by using the 3σ criterion, to determine the statuses in the different switching processes of the substation.
2. The proposed detection method outperforms other fault detection methods. (i) It detects power failure in a small area in a timely manner; (ii) it ignores the critical limitations (poor switching function and limited terminal access rate) of the GSM system in dynamic detection; (iii) it considers all of autocorrelation phenomena in the data collection process. Therefore, the proposed method can comprehensively consider the influence of various factors, and it is easily implemented with better detection results. It is a reliable method for identifying faults.
3. The experimental results herein demonstrate that the results of detection are correct. The method can be used for on-line detection and can support immediate maintenance according to the results of fault detection. It is an effective detection method in support of the safe operation and maintenance of the substation.
4. Although the proposed method is highly adaptable, it is limited by its theoretical assumptions concerning the time series model and the SPC control chart, such as the following. (i) The fitted model does not extract all of the correlation and differences embedded in the original sequence; (ii) the SPC method has two types of discrimination errors, which are false alarms and missed alarms. To overcome the above limitations, attention must be paid by workers in relevant departments and those with knowledge of science and technology.

Finally, future research should address multiple topics to maximize the detection accuracy of the proposed method; to support early warnings, and ultimately to reduce the occurrence of accidents and losses. To improve the applicability and accuracy of the proposed method, some advanced fault detection methods that are used with electrical equipment in other fields could be incorporated into the proposed model. Furthermore, to ensure the stability of the signal transmission process, robust quantization control from the field of nonlinear systems should also be applied.

## Materials and methods

### Autoregressive integrated moving average (ARIMA) model

Proposed by Box et al.^32^, the autoregressive integrated moving average (ARIMA) model is well-known for use in univariate regression. The ARIMA model assumes that the future values in an actual time series, $\left\{ {y_{t} } \right\}_{t = 1}^{N}$, should be a linear combination of past values and past errors, as given by Eq. (3),

3 $$ y_{t} = a_{0} + \beta_{1} y_{t - 1} + \beta_{2} y_{t - 2} + \cdots + \beta_{p} y_{t - p} + \varepsilon_{t} - a_{1} \varepsilon_{t - 1} - a_{2} \varepsilon_{t - 2} - \cdots - a_{q} \varepsilon_{t - q} $$

where $$\beta_{i}$$ and $$a_{i}$$ are coefficients; *p* and *q* are integers from two parts, which are often called the autoregressive polynomial and moving average polynomial, respectively; $$ y_{t}$$ is the actual value at time *t*; $$\varepsilon_{t}$$ is the random error at time *t*, which is theoretically defined as identical and independent distribution (*iid*) and normal distribution.

The difference operator ($\nabla$) is used to solve the non-stationary problem, as defined by Eq. (4),

4 $$ \nabla^{d} y_{t} = \nabla^{d - 1} y_{t} - \nabla^{d - 1} y_{t - 1} $$

ARIMA modelling consists of three processes, which are the estimation of relevant parameters, model verification, and final checking. A useful operator, *B*, is defined to perform the backward shift. Equation (5) defines the *p-*time backwardly shifted actual values, $y_{t}$; Eq. (6) defines the *q*-time backwardly shifted random errors, $\varepsilon_{t}$.

5 $$ B^{1} y_{t} = y_{t - 1} ,\;B^{2} y_{t} = y_{t - 2} , \ldots ,B^{p} y_{t} = y_{t - p} $$

6 $$ B^{1} \varepsilon_{t} = \varepsilon_{t - 1} ,\;B^{2} \varepsilon_{t} = \varepsilon_{t - 2} , \ldots ,\,B^{q} \varepsilon_{t} = \varepsilon_{t - q} $$

Then, the regular autoregressive operator $\varphi_{p} \left( B \right)$ (of order *p*) for the actual values, $y_{t}$, can be further defined as in Eq. (7). Similarly, the regular moving average operator $\psi_{q} \left( B \right)$ (of order *q*) for the random errors, $\varepsilon_{t}$, can be defined by Eq. (8).

7 $$ \varphi_{p} \left( B \right) = 1 - \varphi_{1} B^{1} - \varphi_{2} B^{2} - \cdots - \varphi_{p} B^{p} $$

8 $$ \psi_{q} \left( B \right) = 1 - \psi_{1} B^{1} - \psi_{2} B^{2} - \cdots - \psi_{q} B^{q} $$

Therefore, Eq. (3) can be transformed to Eq. (9),

9 $$ \varphi_{p} \left( B \right)\nabla^{d} y_{t} = C_{0} + \psi_{q} \left( B \right) \varepsilon_{t} $$

Equation (9) is the standard formula for an ARIMA model, and it is often denoted as $ARIMA_{{\left( {p,d,q} \right)}}$ with a non-zero constant, $C_{0}$. For example, the $ARIMA_{{\left( {3,2,1} \right)}}$ model can be specified as Eq. (10),

10 $$ \varphi_{3} \left( B \right)\nabla^{2} y_{t} = C_{0} + \psi_{1} \left( B \right) \varepsilon_{t} $$

Theoretically, the parameters *p*, *d*, and *q*, of an ARIMA model can be estimated by applying the autocorrelation function (ACF) and the partial autocorrelation function (PACF) to the differenced series.

### Seasonal autoregressive integrated moving average (SARIMA) model

Box et al. presented the seasonal ARIMA model (SARIMA model)^32^, whose corresponding process is often denoted as $SARIMA_{{\left( {p,d,q} \right) \times \left( {P,D,Q} \right)}}$. Based on the same theoretical assumption as that made by the ARIMA model, the regression values of an SARIMA model are a linear combination of past values and past errors.

Based on the definitions in previous section, suppose that the actual values series, $\left\{ {y_{t} } \right\}_{t = 1}^{N}$, has a seasonal period length, *S*, in the SARIMA modelling process. Then, the differenced series, $\Delta_{t}$, is defined using a stationary autoregressive moving average process, given by Eq. (11),

11 $$ \Delta_{t} = \left( {1 - B} \right)^{d} \left( {1 - B^{S} } \right)^{D} y_{t} $$

where *d* and *D* both are nonnegative integers.

Then, the $SARIMA_{{\left( {p,d,q} \right) \times \left( {P,D,Q} \right)}}$ model is described by Eq. (12),

12 $$ \varphi_{p} \left( B \right){\Phi }_{P} \left( {B^{S} } \right){\Delta }_{t} = \psi_{q} \left( B \right){\Psi }_{Q} \left( {B^{S} } \right)\varepsilon_{t} \quad t = 1,2, \ldots ,N $$

where *N* is the number of actual values; *B* is the backward shift operator and is defined as Eqs. (5) and (6);$\varphi_{p} \left( B \right)$ is the regular (non-seasonal) autoregressive operator of order *p*, defined by Eq. (7); $\psi_{q} \left( B \right)$ is the regular moving average operator of order *q*, defined by Eq. (8); ${\Phi }_{P} \left( {B^{S} } \right)$ is a seasonal autoregressive operator of order *P*, defined by Eq. (13); ${\Psi }_{Q} \left( {B^{S} } \right)$ is a seasonal moving average operator of order *Q*, defined by Eq. (14); and $\varepsilon_{t}$ is the random error at time *t,* defined as in “Fault detection preparation and implementation” section.

13 $$ {\Phi }_{P} \left( {B^{S} } \right) = 1 - {\Phi }_{1} B^{S} - \cdots - {\Phi }_{P} B^{PS} $$

14 $$ {\Psi }_{Q} \left( {B^{S} } \right) = 1 - {\Psi }_{1} B^{S} - \cdots - {\Psi }_{Q} B^{QS} $$

Based on the above definitions, obviously (i) the parameters *p* and *q* are the orders of autoregression and the moving average, respectively; (ii) the parameters *P* and *Q* are the orders of autoregression and the moving average, respectively, given seasonal length, *S*; and (iii) the parameters *d* and *D* are the orders of the ordinary difference and the seasonal difference, respectively.

In the procedure of the SARIMA model, the orders of difference, *d* and *D*, are estimated first to make the series stationary and to filter out the seasonality. The other values, *p*, *q*, *P*, and *Q,* are estimated from the ACF and PACF of the differenced series. Owing to the non-stationary and periodic characteristics of the substation current data, the $SARIMA_{{\left( {p,d,q} \right) \times \left( {P,D,Q} \right)}}$ model is used.

The parameters (*p* and *q*) for an SARIMA model are determined by observing the censored and trailing of the autocorrelation graph and partial autocorrelation graph of the sequence. In which, “censored” refers to the property that the autocorrelation function (ACF) or partial autocorrelation function (PACF) of time series are all 0 after a certain order (such as PACF of AR)’ the “trailing” is a property that ACF or PACF are not 0 after a certain order (such as the ACF of AR). Model recognition rules are shown in Table 6. The determination of the parameters (*P* and *Q*) for an SARIMA model is followed the same procedure mentioned above under the given seasonal length, *S*.

**Table 6:** Summary of ARMA model recognition rules.

Model	ACF	PACF
AR(*p*)	Tailing	Censored after *p* order
MA(*q*)	Censored after *q* order	Tailing
ARMA(*p*,*q*)	Tailing	Tailing

### Statistical process control

Statistical process control method depends on statistical methods^33^. It analyzes and evaluates an operation process and detects signs of systemic factors using feedback information in a timely manner to maintain the process in a controlled state that is only affected by random factors to ensure quality.

The SPC method is based on the assumptions that if the process is only affected by random factors, then it is in the statistically controlled state (“controlled state”), whereas if it is also controlled by a system factor, then it is in a statistically uncontrolled state (“out-of-control state”). Since the fluctuations in the process exhibit statistical regularity, when the process is in a controlled state, its quality characteristics usually obey a stable random distribution, but when it is in an out-of-control state, the process distribution changes.

The SPC method exploits the statistical regularity of the fluctuations in a process to analyze and control that process. It emphasizes that the process should be conducted in a controlled state. Therefore, the SPC can be used to extract the characteristics of current variation in the switching process behavior of a substation. The method uses statistical information about the current variation sample to judge the process condition, and takes timely measures to reduce the effect of the abnormal factor on the process, to improve the efficiency of the process. The quality of fault detection is thus improved and the detection cost is reduced. The analysis and control capabilities of the control chart not only provide a reliable assessment of the process, but also support an early warning system of monitoring and the prevention of serious failures.

### Singularity discriminating criterion

**Figure 3:**
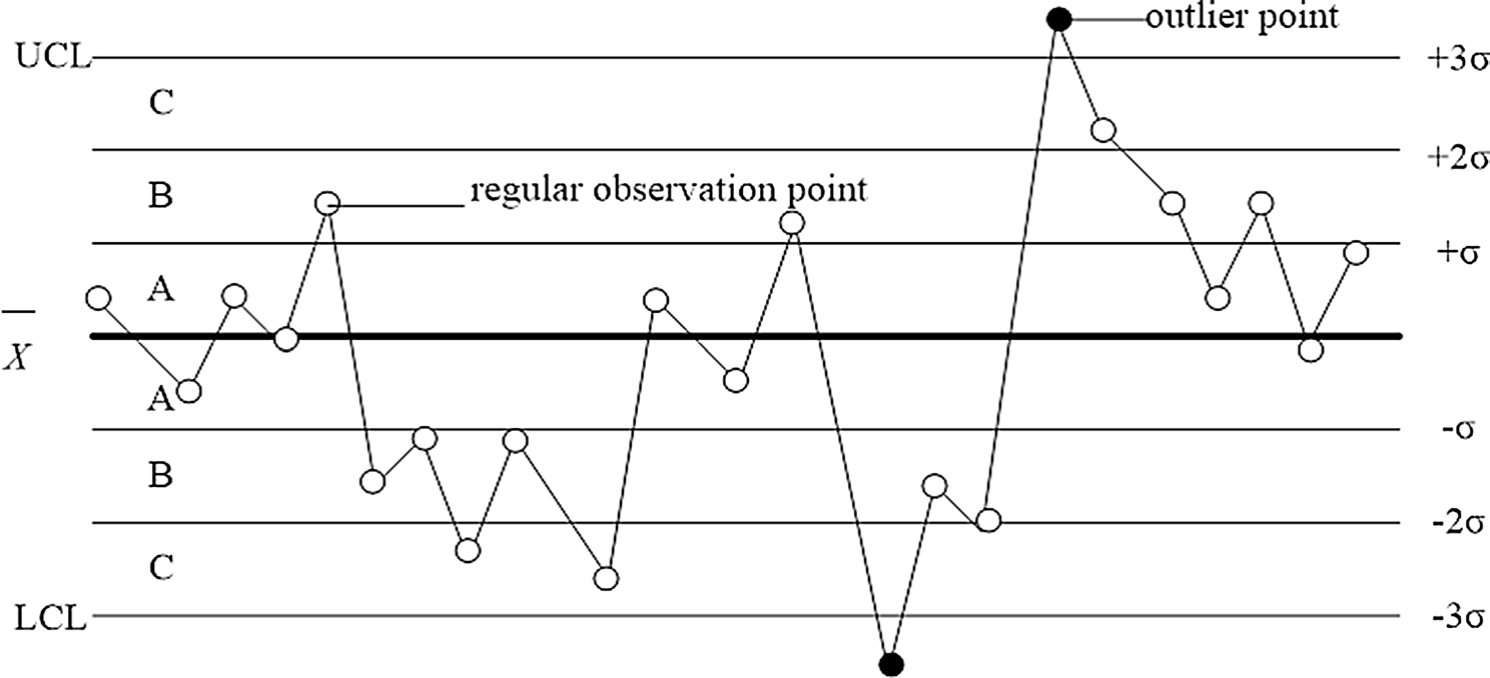
The outlier points and regular points for the singularity discriminating criterion.

The fault discriminating criterion assumes the existence of test data with random errors and a calculated standard deviation, from which an interval with a certain probability is determined. If the duration of an error exceeds the determined interval, it is not a random error but a singular error. In this paper, the 3σ criterion (presented in Fig. 3) is applied to conduct the singularity discriminant detection. If an error (as shown as a point in Fig. 3) on the control chart is between the upper control limit (UCL) and the lower control limit (LCL), then it is regarded as the qualified point (inlier point), and the substation has no fault. In contrast, if it is a point of failure (outlier), then the substation is in a state of failure.

Comparing to 2σ criterion and 4σ criterion, 3σ criterion is applicable when dealing with many data sets, and it is generally applied when the number of measurements is sufficiently large (i.e., $n \ge 30$). Thus, 3σ is taken to be as the limit error, and the 3σ criterion is the most common and the simplest criterion for gross error. In order to minimize the total loss caused by the two types of errors, the centre line of the upper and lower limits of the control chart is $\pm \;3\sigma$. In this paper, due to hundreds of testing times, 3σ criterion is employed.

In this, paper, outlier points principally imply that the associated faults are caused by (i) improper use of new operators, leading to machine failure; (ii) voltage instability; (iii) fatigue of the system; (iv) changes in detection methods or calculation standards; and even (v) calculation errors or measurement errors. Inlier points often imply that the substation is without any fault and that any of the following situations may be occurred, (a) the equipment or tools are wearing out gradually; (b) the maintenance level is gradually falling; (c) the skills of the operator are gradually improving.

### Model for evaluating high-voltage circuit breaker status

As is well known, circuit breakers are critical devices at a substation. Owing to their frequent operation, these large mechanical parts commonly fail. It is very important to monitor the mechanical status of the circuit breaker and diagnose the health condition to prevent failure of the substation.

**Figure 4:**
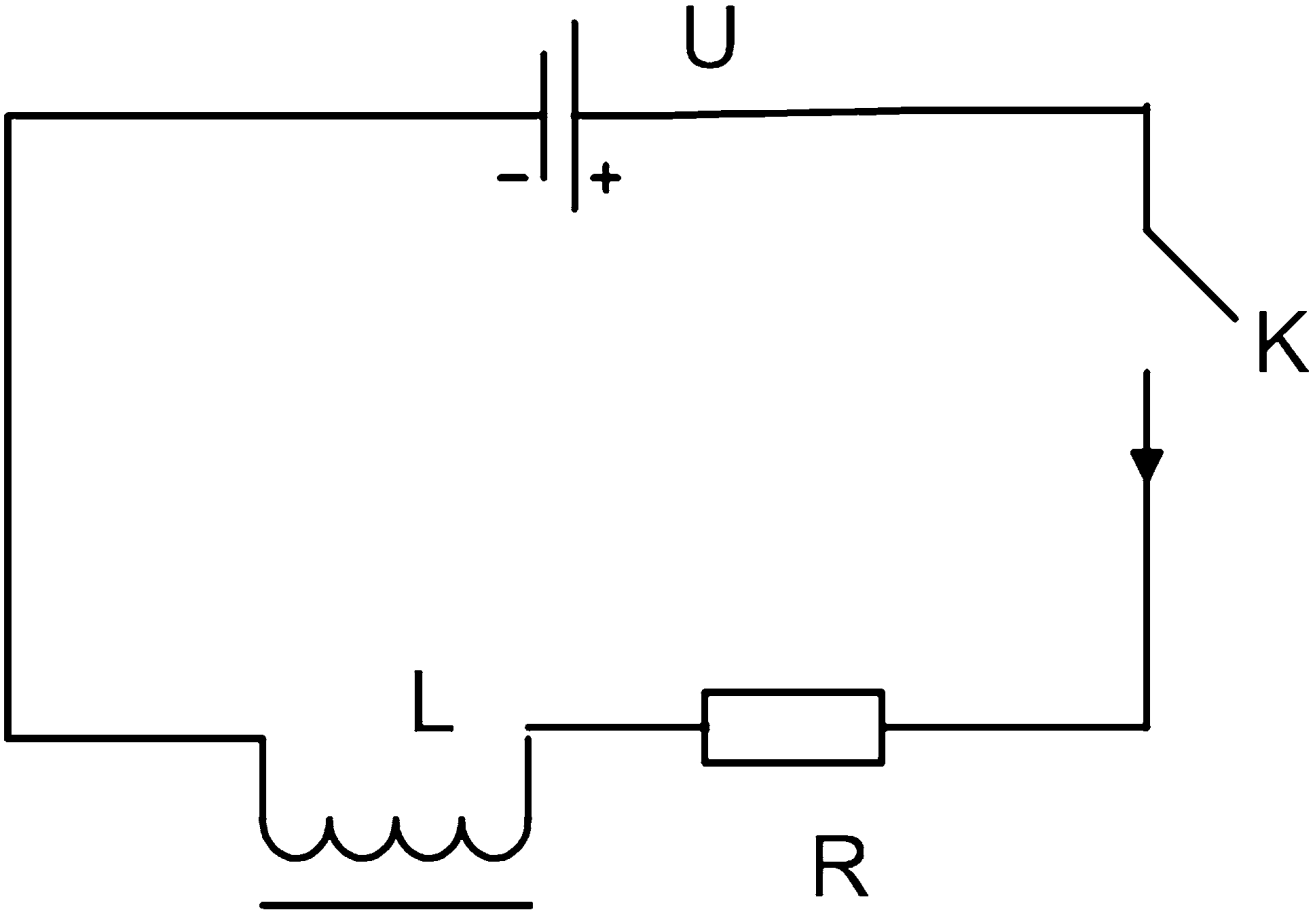
The DC power supply of the circuit breaker.

The high-voltage circuit breaker is based on an electromagnet with DC and the current waveform of the DC electromagnetic coil contains important information for diagnosing mechanical faults. Figure 4 presents the DC power supply of such a circuit breaker.

In Fig. 4, *U* represents the DC power supply voltage; *K* represents the distribution switch; *R* represents the coil resistance; *L* is the coil inductance; and *i* is the current in the coil. The value of *L* depends on the sizes of the coil and the iron core yoke; it is also closely related to the iron stroke, *S*, which is the path along which the iron core moves upward. *L* increases with *S*.

If the iron core is not saturated, then *L* is independent of *i*. After the switch *K* is closed in the circuit, the DC power supply voltage, *u*, is as given by Eq. (15),

15 $$ u = iR + \left( {\frac{dw}{{dt}}} \right) $$

where *w* represents the number of magnetic linkages of the coil, and $w = i \times L$. Therefore, Eq. (15) can be transformed to Eq. (16),

16 $$ u = iR + \left( {\frac{diL}{{dt}}} \right) = iR + L\left( {\frac{di}{{dt}}} \right) + i\left( {\frac{dL}{{dS}}} \right)\left( {\frac{dS}{{dt}}} \right) $$

where $\left( {\frac{dL}{{dS}}} \right)$ represents the slope of the curve at *S*; $\left( {\frac{dS}{{dt}}} \right)$ represents the speed of the iron core, and is denoted as *v*. Accordingly, Eq. (16) can be further transformed to Eq. (17),

17 $$ u = iR + L\left( {\frac{di}{{dt}}} \right) + i\left( {\frac{dL}{{dS}}} \right)v $$

**Figure 5:**
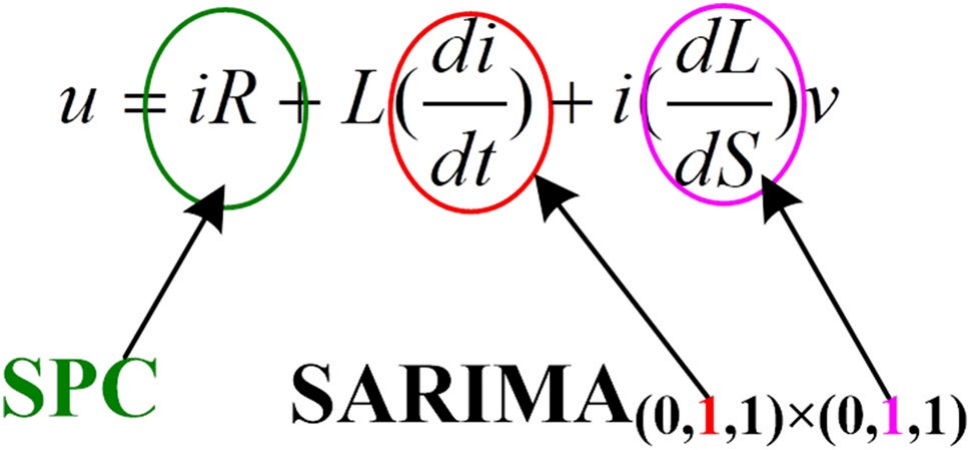
The correspondent relationships among the DC power supply voltage, SPC, and SARIMA model.

Equation (17) contains two derivatives that are related to *i*, and a $SARIMA_{{\left( {0,1,1} \right) \times \left( {0,1,1} \right)}}$ model, as presented in Fig. 5.

The $\frac{di}{{dt}}$ term corresponds to the first-order difference of the $SARIMA_{{\left( {0,1,1} \right) \times \left( {0,1,1} \right)}}$ model without seasonal effects. The $\frac{di}{{dt}}$ term refers to the dynamic change of the inherent principal regulation of the current, which is the most stable form of regulation. If the $\frac{di}{{dt}}$ term becomes inconsistent with the $SARIMA_{{\left( {0,1,1} \right) \times \left( {0,1,1} \right)}}$ model, then a serious fault has occurred such that the system cannot be repaired. The corresponding parameters of the $SARIMA_{{\left( {0,1,1} \right) \times \left( {0,1,1} \right)}}$ model without seasonal effects are $p = 0$ and $q = 1$, which indicate that the range of feature points exhibits no self-similarity, but the inertia of the system causes the sliding average effect. It further explains the simplicity and interpretability of the proposed approach.

The $\frac{dL}{{dS}}$ term corresponds to the first-order difference of the $SARIMA_{{\left( {0,1,1} \right) \times \left( {0,1,1} \right)}}$ model with seasonal effects. Although the $\frac{dL}{{dS}}$ term is not directly caused by the current, it has a differential relationship with the current, which is generally stable. If the $\frac{dL}{{dS}}$ term becomes inconsistent with the $SARIMA_{{\left( {0,1,1} \right) \times \left( {0,1,1} \right)}}$ model, then the fault becomes more obvious, and can be repaired.

The $iR$ term implies that the characteristics of current variation will have a certain degree of deviation. The deviations are demonstrated as a sub-health status that is caused by fatigue of the components. Such a status can be further detected using the SPC control chart. If the $iR$ item is an inlier point, then the status is good; if it is an outlier point, then the system has a sub-health status, and so must be repaired to extend its service life.

Accurately describing the complex behaviors of equipment during its operation using a traditional univariate SPC model is difficult because the model does not consider correlation among variables. The multivariate model is more complicated with more difficult variable selection and information acquisition. This proposed multivariate model can effectively decompose correlations. The univariate SPC model can be easily and effectively implemented after the remaining part has been extracted, so, it can overcome the shortcomings of the traditional method.

For example, the ACTAS P14 (version 2.5; below is briefed as ACTAS P14) is a real-world system for testing medium to high voltage switches. During the testing period, the switching device is connected to the testing system to measure the important electrical and mechanical parameters and then used for analysis. The ACTAS P14 is a master/slave system consisting of a testing instrument and a standard computer with the operating and analysis software ACTAS P14. Both are connected via a serial interface (RS232) or USB interface. The computer is the main control testing instrument to control the testing instrument (from the system). After the configuration, the testing instrument is used to perform the testing action. The testing object action is recorded in real time, and the computer reads the recorded data for analysis. The ACTAS P14 has 14 analog measurement inputs (8 analog sensors and 6 digital sensors) and up to 66 binary measurement inputs, plus more than 10 control output interfaces. The control output is used to test the object for switching operations. The action of the testing object is reported by the signal in real time and analyzed on the computer. Except for the ON/OFF switch, the testing instrument itself has no parts to be operated, so all the data operation and control of our entire process is through the standard ACTAS P14 computer software.

## Analysis and characteristics of substation switching process

In this paper, we collect large number of the split closing coil current characteristic parameter from the original circuit breaker, the total of 4,800 points for each separation process, sampling frequency is 53.3(= 4,800/90) Hz, that is, sampling about 53 points per second. Then, a sample of the mechanical characteristics from the original circuit breaker could be generated.

Furthermore, the preliminary statistical analysis and processing are carried out to obtain the mentioned characteristic parameter sample of the circuit breaker, the status regulation of the circuit breaker is then studied, and finally, the health status of the circuit breaker could be tested and evaluated.

### SARIMA model analysis of substation switching process

The proposed SARIMA model is based on current data from a substation switching process between 1st January 2017 and 30th June 2017. The relevant specific sub-processes are as follows.

#### Extracting current characteristic data in switching process and plotting process variation diagram of quality characteristics

Historical data are extracted from a data set obtained at a substation, and all power plant switching processes are analyzed. The time series diagram is provided as the process variation diagram of the quality characteristics. A current value occurs at different times in different cycles, however, in each cycle, three points of extreme current exist under normal fluctuations, and their values are all stable. Therefore, the test of the abnormality of each switching process can be converted into the test of the three extreme points of the current.

**Figure 6:**
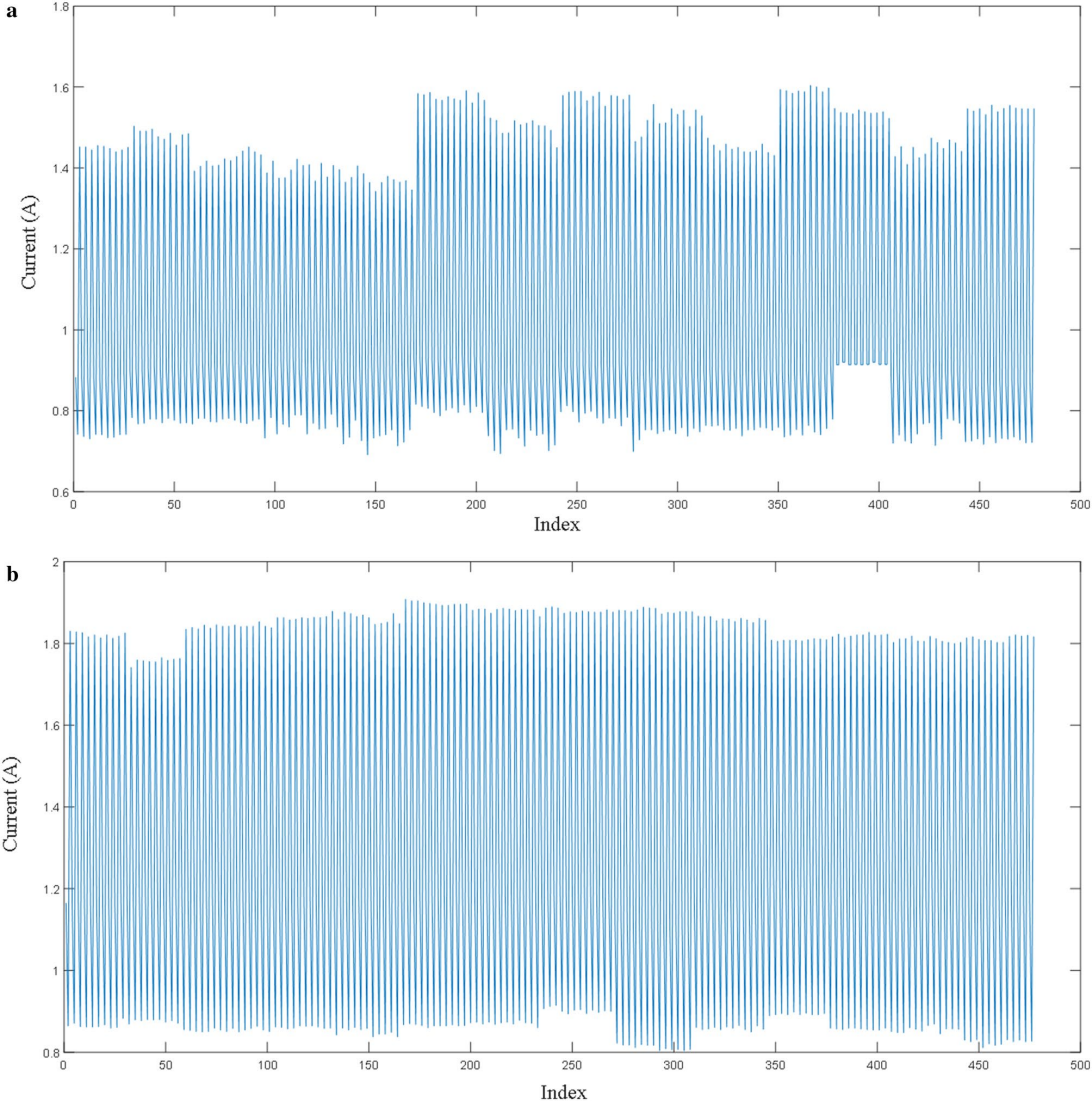
(**a**) Open process time series. (**b**) Close process time series.

The substation switching process is divided into two sub-processes, which are the open process and the close process. The original current data for open process and the close process are demonstrated in Fig. 6a,b, respectively. First, the time series diagram is obtained in the open process. Second, based on the current curves, the statuses of the three extreme points in each cycle, extracted the data from various power stations are considered, and then the different operator is applied to a sequence with non-stationary and seasonal characteristics to generate a stationary sequence. The two operations in the open process are similar to those in the closed process.

#### Stabilizing and modeling SARIMA model using autocorrelation and partial autocorrelation diagrams

**Figure 7:**
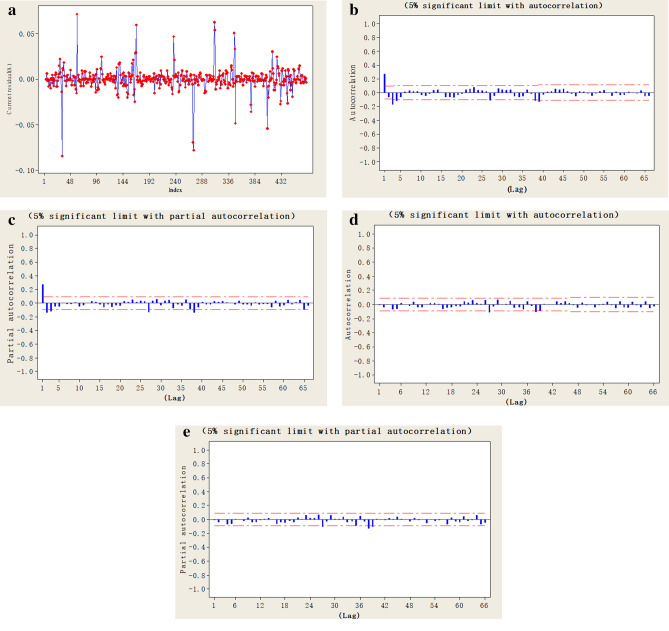
(**a**) The sequence diagram after the difference with 1st order for the open process (Calculated by JMP10.0 (SAS Institute Inc.)). (**b**) The autocorrelation diagram from the 1st order differencing process (Calculated by JMP10.0 (SAS Institute Inc.)). (**c**) The partial autocorrelation diagram from the 1st order differencing process (Calculated by JMP10.0 (SAS Institute Inc.)). (**d**) The residual autocorrelation diagram for the open process (Calculated by JMP10.0 (SAS Institute Inc.)). (**e**) The residual deviation autocorrelation diagram for the open process (Calculated by JMP10.0 (SAS Institute Inc.)).

The received sequence diagram after the difference with 1st order in Fig. 7a demonstrates that it is basically in a stationary state. Moreover, the autocorrelation and partial autocorrelation diagrams obtained during the 1st differencing process, as presented in Fig. 7b,c, are used to determine the most appropriate model. For sequences with non-stationary and seasonal characteristics, as mentioned above, the $SARIMA_{{\left( {p,d,q} \right) \times \left( {P,D,Q} \right)}}$ model is used. Similarly, the parameters (*p*, *d*, *q*) and (*P*, *D*, *Q*) are determined using the autocorrelation and partial autocorrelation diagrams. The results (Fig. 7a–e) of this process are calculated using the statistical software JMP10.0, provided by the SAS Institute Inc.

The *p*-value of the fitting results, shown in Table 6, indicates that the model closely fits the original data. The autocorrelation and partial autocorrelation diagrams of the residuals in Fig. 7d,e, respectively, reveal that no cylinder falls outside the upper and lower red limits. The correlation among data was eliminated at a significance level α = 0.05.

After the current data for the substation switching processes have been extracted and sorted, the $ARIMA_{{\left( {0,1,1} \right)}}$ model is used to perform the pre-fitting of the extracted current data. If the pre-fitting results are not satisfactory, then the substation is obviously under the failure status. At this moment, the system has a serious fault, and is difficult to recover. If the pre-fitting results are satisfactory, they are further analyzed using the $SARIMA_{{\left( {p,d,q} \right) \times \left( {P,D,Q} \right)}}$ model to evaluate the received residual error.

Two $SARIMA_{{\left( {0,1,1} \right) \times \left( {0,1,1} \right)}}$ and $SARIMA_{{\left( {0,1,1} \right) \times \left( {1,1,1} \right)}}$ models are established using the above pre-fitted results, and the model that better explains the characteristics of the switching process is determined. Tables 7 and 8 presents statistical analyses of these two SARIMA models, respectively. Tables 7 and 8 indicate that the $SARIMA_{{\left( {0,1,1} \right) \times \left( {1,1,1} \right)}}$ model yields a smaller *p*-value under the level of significance ${\upalpha } = 0.05$, revealing that the $SARIMA_{{\left( {0,1,1} \right) \times \left( {0,1,1} \right)}}$ model fits better than the $SARIMA_{{\left( {0,1,1} \right) \times \left( {1,1,1} \right)}}$ model. Given the selection criterion of models, the $SARIMA_{{\left( {0,1,1} \right) \times \left( {0,1,1} \right)}}$ model with the favorable fitting and easy implementation is selected as the most suitable model to demonstrate the characteristics of the switching process.

**Table 7:** The statistical analysis of the $SARIMA_{{\left( {0,1,1} \right) \times \left( {0,1,1} \right)}}$ model.

Models	Coefficients	Standard deviation of coefficients	*p*-value	Residual error (MS_2_)
SMA*	0.511	0.0459	0.000	0.00072
MA**	0.719	0.0554	0.000	

*SMA model implies the $SARIMA_{{\left( {0,1,1} \right) \times \left( {0,1,1} \right)}}$ model.

**MA model implies the $ARIMA_{{\left( {0,1,1} \right)}}$ model.

**Table 8:** The statistical analysis of the $SARIMA_{{\left( {0,1,1} \right) \times \left( {1,1,1} \right)}}$ model.

Models	Coefficients	Standard deviation of coefficients	*p*-value	Residual error (MS_2_)
SAR*	− 0.179	0.0746	0.017	0.000677
SMA**	0.850	0.0559	0.000	
MA***	− 0.189	0.0962	0.050	

*SAR model implies the $SARIMA_{{\left( {0,1,1} \right) \times \left( {1,1,1} \right)}}$ model.

**SMA model implies the $SARIMA_{{\left( {0,1,1} \right) \times \left( {0,1,1} \right)}}$ model.

***MA model implies the $ARIMA_{{\left( {0,1,1} \right)}}$ model.

### SARIMA model for detecting faults of substation equipment

**Figure 8:**
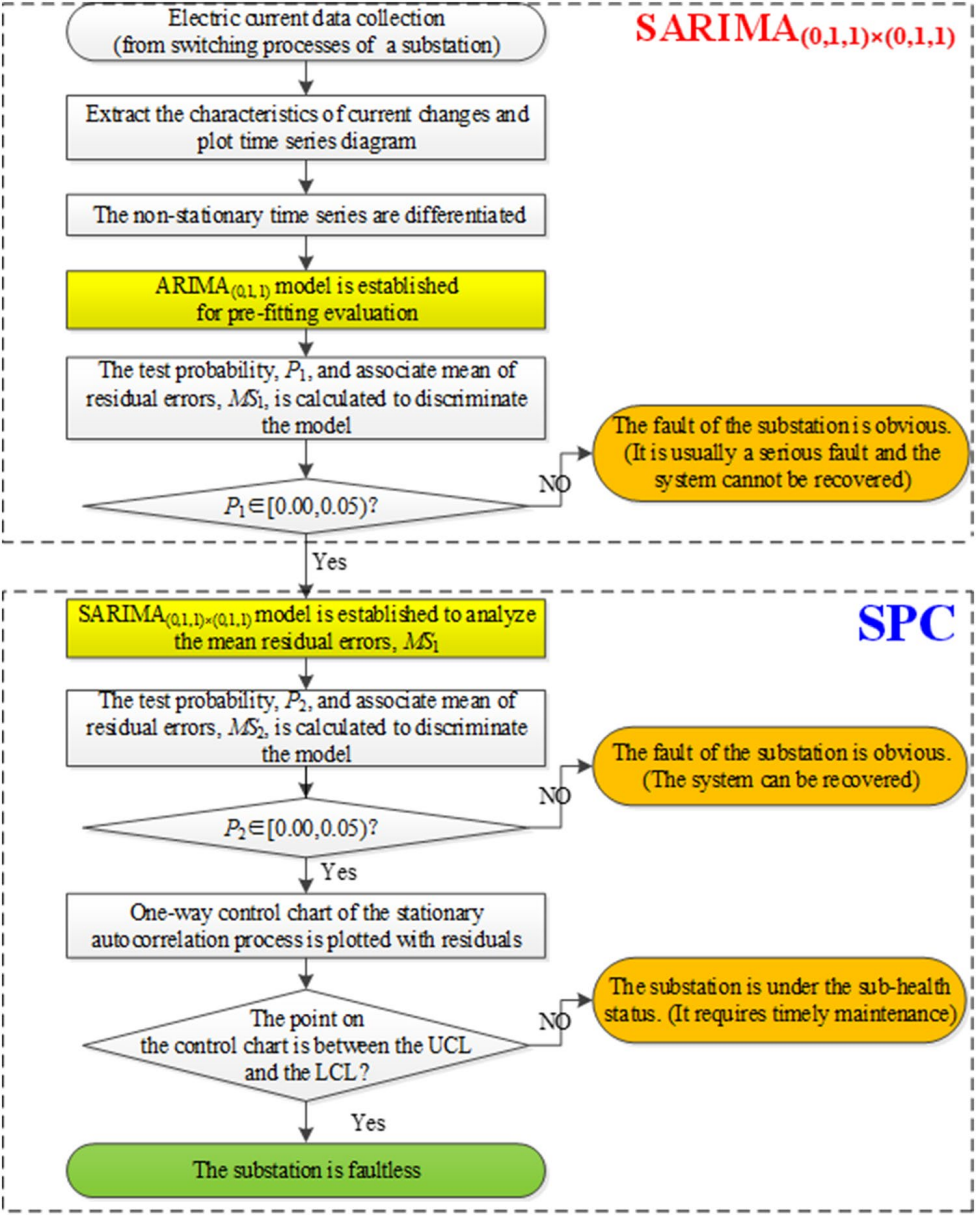
The flow chart of the proposed SARIMA-SPC model.

Based on the statistical analysis of the SARIMA model mentioned in previous section, the principal characteristics of the model are extracted. Accordingly, the SPC method can be feasibly hybridized with the SARIMA model to detect faults of substation equipment. The implementation steps are as follows, and the associated flow chart is presented in Fig. 8.

#### Step 1 extract the current variation and plot time series diagram

**Figure 9:**
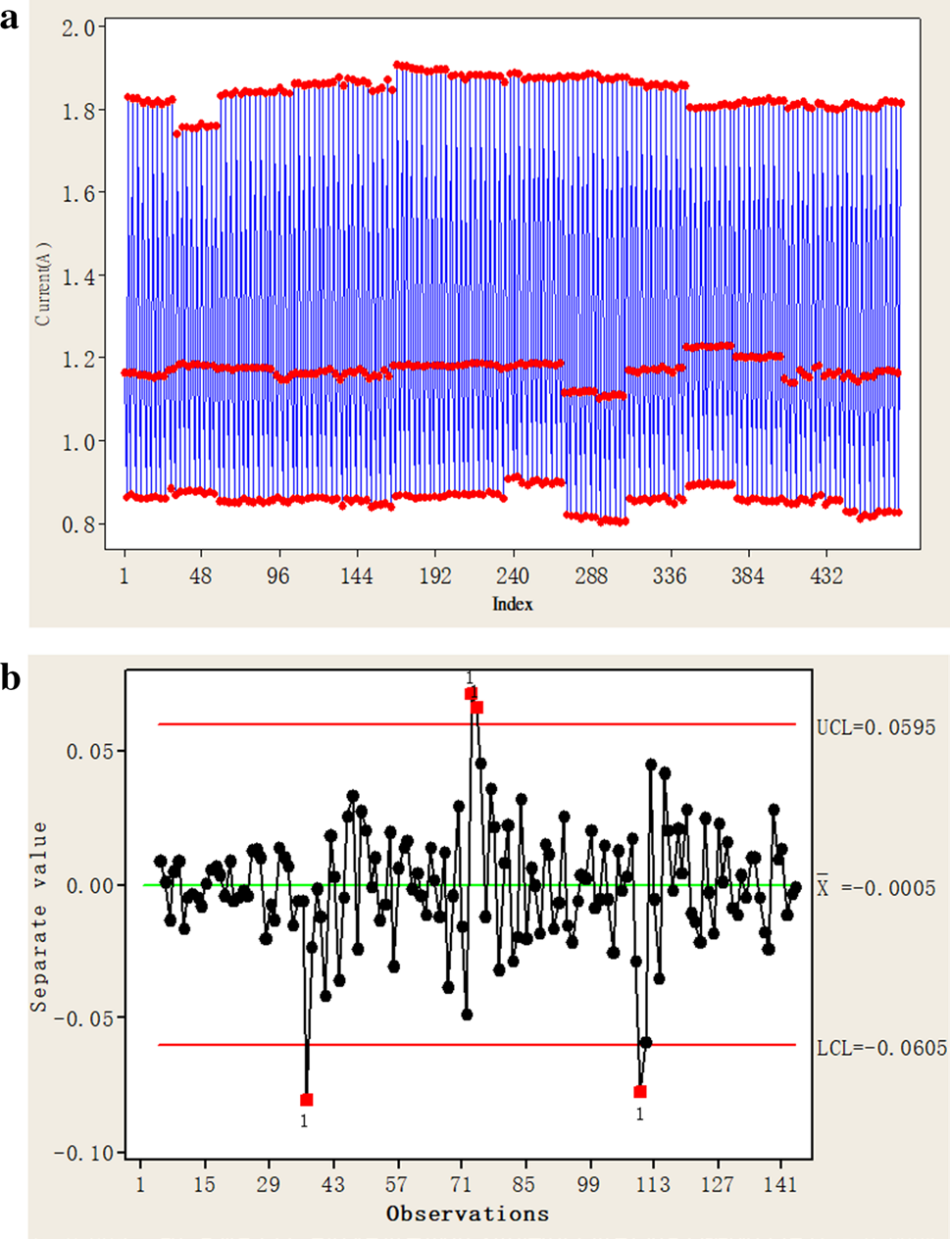
(**a**) The diagram of the employed time series (Calculated by Minitab 16.0 version (Minitab Inc.)). (**b**) The one-way control chart. (Calculated by Minitab 16.0 version (Minitab Inc.)).

Extract the changing characteristics of the current variation in the switching process of the substation. Collect the sampling data points using a certain sampling interval, and identify the extreme points; plot the time series diagram of these sampling points, as presented in Fig. 9a. These results (Fig. 9a,b) of this process are calculated using the statistical software Minitab version 16.0 by Minitab Inc.

#### Step 2 preliminary failure detection

Difference the non-stationary time series in *Step 1*, and perform the pre-fitting process by using the $ARIMA_{{\left( {p,d,q} \right)}}$ model to obtain the correlated residual error, $MS_{1}$, and the associated probability, $P_{1}$, which is used to evaluate the performance of the estimated ARIMA model. Only if $P_{1} \notin \left[ {0.00, 0.05)} \right.$ is the pre-fitting performance not satisfactory, indicating that the substation is obviously under the failure status. And, at this moment, the system has serious faults, and its recovery is likely to be difficult, such as in cases of a voltage transformer fault, a current transformer fault, a capacitor fault, the refusal of a circuit breaker to close, bus failure, and others. In contrast, if $P_{1} \in \left[ {0.00, 0.05)} \right.$, then the pre-fitting effect is satisfactory and should be further analyzed by using the $SARIMA_{{\left( {p,d,q} \right) \times \left( {P,D,Q} \right)}}$ model to obtain residual error.

#### Step 3 advanced failure detection

Based on the residual error, $MS_{1}$, obtained in *Step 2*, the $SARIMA_{{\left( {p,d,q} \right) \times \left( {P,D,Q} \right)}}$ model is used for fitting, and to obtain the correlated residual error, $MS_{2}$, and the associated probability, $P_{2}$, which is in turn used to evaluate the performance of the estimated SARIMA model. Only if $P_{2} \notin \left[ {0.00, 0.05)} \right.$ is the fitting performance not satisfactory, indicating that the substation under the failure status more obviously. At that moment, the faults suffered by the system, including resonance, the system mixed line, the porcelain bottle flicker, and other, can be recovered. If $P_{2} \in \left[ {0.00, 0.05)} \right.$, then the fitting effect is satisfactory, and go to the next step for further detection.

#### Step 4 singularity discriminant detection using the 3σ criterion

Based on the residual error, $MS_{2}$, obtained in *Step 3*, plot a one-way control chart of the stationary autocorrelation process, and perform the singularity discriminant detection using the 3σ criterion. If the point on the control chart is between the UCL and the LCL, then it is regarded as the qualified point (inlier point), and the substation has no fault. Otherwise, it is a point of failure (outlier point). The substation is in a sub-health status as a result of a stuck mechanism or poor contact at the line head, for example, and requires timely maintenance.

Implementing the above steps to process the test samples yields the test results in Fig. 9b.

The proposed method completely considers the change process of state space and time dimension. It not only overcomes the problem of static detection delay, but also easily extracts the fault signal characteristics of dynamic changes. If there are changes in the data, the order and parameters of the model can be adjusted according to the principle of order determination.
